# Supplementary material for: Identification and functional analysis of non-coding regulatory small RNA FenSr3 in Bacillus amyloliquefaciens LPB-18
Source: PeerJ. 2023 May 15;11:e15236. doi: 10.7717/peerj.15236 (PMC10194069; doi:10.7717/peerj.15236)
Supplement: Supplemental Information 4 [file peerj-11-15236-s004.zip › KO/CK-vs-T1_map/map04666.html]

KEGG PATHWAY: Fc gamma R-mediated phagocytosis - Reference pathway


|  |  |
| --- | --- |
| **Fc gamma R-mediated phagocytosis - Reference pathway** |  |

[
Pathway menu
| Organism menu
| Pathway entry
| Show description
| User data mapping
]

Phagocytosis plays an essential role in host-defense mechanisms through the uptake and destruction of infectious pathogens. Specialized cell types including macrophages, neutrophils, and monocytes take part in this process in higher organisms. After opsonization with antibodies (IgG), foreign extracellular materials are recognized by Fc gamma receptors. Cross-linking of Fc gamma receptors initiates a variety of signals mediated by tyrosine phosphorylation of multiple proteins, which lead through the actin cytoskeleton rearrangements and membrane remodeling to the formation of phagosomes. Nascent phagosomes undergo a process of maturation that involves fusion with lysosomes. The acquisition of lysosomal proteases and release of reactive oxygen species are crucial for digestion of engulfed materials in phagosomes.

|  |  |  |
| --- | --- | --- |
| Reference pathway | 184% 150% 122% 100% 82% 67% 55% | 图片下载 |
